# Metabolic causes of liver disease among adults living with HIV from low‐ and middle‐income countries: a cross‐sectional study

**DOI:** 10.1002/jia2.26238

**Published:** 2024-04-02

**Authors:** Marie Kerbie Plaisy, Albert K. Minga, Gilles Wandeler, Gad Murenzi, Niharika Samala, Jeremy Ross, Alvaro Lopez, Ephrem Mensah, Renée de Waal, Mark H. Kuniholm, Lameck Diero, Sonali Salvi, Rodrigo Moreira, Alain Attia, Ardele Mandiriri, Fabienne Shumbusho, Suzanne Goodrich, Dhanushi Rupasinghe, Paola Alarcon, Fernanda Maruri, Hugo Perrazo, Antoine Jaquet, the Sentinel Research Network of the International epidemiology Databases to Evaluate AIDS

**Affiliations:** ^1^ University of Bordeaux, National Institute for Health and Medical Research (INSERM) UMR 1219, Research Institute for Sustainable Development (IRD) EMR 271, Bordeaux Population Health Centre Bordeaux France; ^2^ Blood Bank Medical Centre, the HIV care clinic of the National Blood Transfusion Centre Abidjan Côte d'Ivoire; ^3^ Department of Infectious Diseases Bern University Hospital University of Bern Bern Switzerland; ^4^ Research for Development (RD Rwanda) and Rwanda Military Hospital Kigali Rwanda; ^5^ Department of Medicine School of Medicine Indiana University Indianapolis Indiana USA; ^6^ TREAT Asia/amfAR – The Foundation for AIDS Research Bangkok Thailand; ^7^ Departamento de Infectología Instituto Nacional de Ciencias Médicas y Nutrición Salvador Zubirán Mexico City Mexico; ^8^ NGO Espoir‐Vie Togo Lomé Togo; ^9^ Centre for Infectious Disease Epidemiology and Research University of Cape Town Cape Town South Africa; ^10^ Department of Epidemiology and Biostatistics University at Albany State University of New York Rensselaer New York USA; ^11^ Department of Medicine School of Medicine College of Health Sciences Moi University Eldoret Kenya; ^12^ Byramjee Jeejeebhoy Government Medical College Pune India; ^13^ Evandro Chagas National Institute of Infectious Diseases‐Oswaldo Cruz Foundation (INI/FIOCRUZ) Rio de Janeiro Brazil; ^14^ University Hospital of Yopougon Abidjan Côte d'Ivoire; ^15^ Newlands Clinic Harare Zimbabwe; ^16^ The Kirby Institute UNSW Sydney Kensington New South Wales Australia; ^17^ Department of Medicine Division of Infectious Diseases Vanderbilt University Medical Center Nashville Tennessee USA

**Keywords:** antiretroviral therapy, HIV acquisition, liver disease, liver fibrosis, low‐ and middle‐income countries, metabolic disorders

## Abstract

**Introduction:**

Liver disease is a leading cause of morbidity and mortality among persons living with HIV (PLHIV). While chronic viral hepatitis has been extensively studied in low‐ and middle‐income countries (LMICs), there is limited information about the burden of metabolic disorders on liver disease in PLHIV.

**Methods:**

We conducted a cross‐sectional analysis of baseline data collected between October 2020 and July 2022 from the IeDEA‐Sentinel Research Network, a prospective cohort enrolling PLHIV ≥40 years on antiretroviral treatment (ART) for ≥6 months from eight clinics in Asia, Americas, and central, East, southern and West Africa. Clinical assessments, laboratory testing on fasting blood samples and liver stiffness measurement (LSM)/controlled attenuation parameter (CAP) by vibration‐controlled transient elastography were performed. Multivariable logistic regression models assessed factors associated with liver fibrosis (LSM ≥7.1 kPa) and steatosis (CAP ≥248 dB/m). Population attributable fraction (PAF) of each variable associated with significant liver fibrosis was estimated using Levin's formula.

**Results:**

Overall, 2120 PLHIV (56% female, median age 50 [interquartile range: 45−56] years) were included. The prevalence of obesity was 19%, 12% had type 2 diabetes mellitus (T2DM), 29% had hypertension and 53% had dyslipidaemia. The overall prevalence of liver fibrosis and steatosis was 7.6% (95% confidence interval [CI] 6.1−8.4) and 28.4% (95% CI 26.5−30.7), respectively, with regional variability. Male sex at birth (odds ratio [OR] 1.62, CI 1.10−2.40), overweight/obesity (OR = 2.50, 95% CI 1.69−3.75), T2DM (OR 2.26, 95% CI 1.46−3.47) and prolonged exposure to didanosine (OR 3.13, 95% CI 1.46−6.49) were associated with liver fibrosis. Overweight/obesity and T2DM accounted for 42% and 11% of the PAF for liver fibrosis, while HBsAg and anti‐HCV accounted for 3% and 1%, respectively. Factors associated with steatosis included overweight/obesity (OR 4.25, 95% CI 3.29−5.51), T2DM (OR 2.06, 95% CI 1.47−2.88), prolonged exposure to stavudine (OR 1.69, 95% CI 1.27−2.26) and dyslipidaemia (OR 1.68, 95% CI 1.31−2.16).

**Conclusions:**

Metabolic disorders were significant risk factors for liver disease among PLHIV in LMICs. Early recognition of metabolic disorders risk factors might be helpful to guide clinical and lifestyle interventions. Further prospective studies are needed to determine the causative natures of these findings.

## 1 INTRODUCTION

With the expanded coverage of antiretroviral treatment (ART), AIDS‐related mortality among persons living with HIV (PLHIV) has substantially decreased, resulting in a growing burden of non‐AIDS comorbidities, including liver diseases [1, 2, 3]. In high‐income countries, liver disease is a leading cause of morbidity and mortality among PLHIV on ART, with well‐recognized risk factors, including hepatitis B virus (HBV) and hepatitis C virus (HCV) infections, alcohol use and metabolic disorders, such as type 2 diabetes mellitus (T2DM) and obesity, which are established main drivers for liver steatosis [3, 4]. In low‐ and middle‐income countries (LMICs), HBV and HCV infections have been reported as the leading causes of liver disease [5, 6]. However, the prevalence of metabolic diseases is increasing in many LMICs, linked to changes in dietary practices and lower physical activity [7].

In high‐income countries, liver steatosis has increased significantly among PLHIV, and is often secondary to the metabolic syndrome and its components (T2DM, hypertension and dyslipidaemia), and mitochondrial damage leading to insulin resistance and dyslipidaemia [8, 9]. Furthermore, some ART drugs, especially some first‐generation ART drugs, could induce lipolysis and mitochondrial dysfunction, leading to steatosis [10]. Previous studies have highlighted the hepatotoxicity of these drugs, with potentially persisting effects even after withdrawal [11, 12]. However, data on liver damage due to cumulative exposure to these older ART drugs remain scarce, especially in LMICs.

Metabolic dysfunction‐associated steatotic liver disease, defined as the presence of steatosis in the absence of excess alcohol consumption and other liver disease, and alcoholic‐associated liver disease are the most common causes of liver steatosis [13]. A proportion of the undiagnosed and untreated liver steatosis can progress to liver fibrosis, cirrhosis, liver failure and hepatocellular carcinoma, with an increased mortality [14]. Liver steatosis is, therefore, a major concern that requires early recognition of persons at risk of liver fibrosis to prevent liver‐related complications. Non‐invasive tools such as vibration‐controlled transient elastography (VCTE) using FibroScan^®^ are increasingly used to measure both liver fibrosis and steatosis [15, 16].

In LMICs, HBV active ART and curative HCV treatments are increasingly available for PLHIV. We, therefore, hypothesize that metabolic disorders have become a major contributor to liver disease among PLHIV. So far, studies that reported factors associated with liver fibrosis among PLHIV in LMICs have been hampered by a lack of common standardized measurement and were mainly focused on HBV or HCV co‐infection [17, 18, 19, 20]. In this study, we aim to estimate the prevalence of significant liver fibrosis and steatosis, and the contributing role of metabolic disorders on these liver outcomes among adults living with HIV on ART in LMICs.

## 2 METHODS

### 2.1 Design and population

We conducted a cross‐sectional analysis of data at enrolment into the Sentinel Research Network (SRN), an ongoing prospective cohort nested in the International epidemiology Databases to Evaluate AIDS (IeDEA) consortium [21]. Participants enrolled in SRN were PLHIV aged ≥40 years on ART ≥6 months attending one of eight HIV clinics in six IeDEA regions (https://www.iedea.org/): Asia‐Pacific (India), central/southern America (CCASAnet) (Brazil, Mexico), central Africa (Rwanda), East Africa (Kenya), southern Africa (Zambia) and west Africa (Côte d'Ivoire, Togo). Participants were recruited during their routine visit using two sampling approaches: systematic random sampling for sites with a large number of eligible PLHIV, or consecutive enrolment for those with a small number of eligible PLHIV. Participants who agreed to participate in the study were scheduled for the baseline visit that included the collection of demographic information, anthropometry, as well as laboratory tests, and VCTE.

### 2.2 Study measurements

Standardized questionnaires were used to collect socio‐demographic information including sex at birth, age, country of residence and marital status. Alcohol use disorder was assessed using the Alcohol Use Disorders Identification Test (AUDIT). The AUDIT questionnaire contains 10 items, each scored from 0 to 4 points, giving a maximum total score of 40 points. An AUDIT score ≥8 in males and ≥7 in females was considered hazardous alcohol use [22]. Measured weight and height were used to calculate body mass index (BMI) = weight (kg)/[height (m)]^2^. BMI from 25 to 29.9 kg/m^2^ and ≥30 kg/m^2^ were used to define overweight and obesity, respectively. Waist and hip circumference were also measured, and the presence of central obesity was defined with waist circumference (WC) ≥94 cm for males and ≥80 cm for females in sub‐Saharan African participants; and WC ≥90 cm for males and ≥80 cm for females in Asian and American participants [23]. Blood pressure (BP) was measured thrice using an automated cuff. Hypertension was defined as mean diastolic BP ≥90 mmHg AND/OR systolic BP ≥140 mmHg or prescribed anti‐hypertension medication [24].

Fasting blood glucose and glycosylated haemoglobin (HbA1c) were measured; T2DM was defined as plasma glucose ≥ 7.0 mmol/l or HbA1c ≥ 6.5% or history of diabetes treatment [25]. A lipid panel was also performed to define dyslipidaemia (plasma levels of total cholesterol≥6.20 mmol/l, triglycerides>2.25 mmol/l, LDL>4.13 mmol/l and HDL<1.03 mmol/l for males or <1.29 mmol/l for females) [26]. Co‐infections with HBV and HCV were systematically assessed using rapid diagnostic tests on participants’ blood samples (Determine®, SD Bioline® or Abon® for HBs antigen and Oraquick® for anti‐HCV antibodies). Those with positive rapid diagnostic tests underwent viral load quantification of HBV‐DNA and/or HCV‐RNA by polymerase chain reaction. Aspartate aminotransferase (AST) and alanine aminotransferase (ALT) were measured for all participants with upper limit of normal (ULN) values of 40 IU/l [27, 28]. HIV viral load and CD4 cell counts were also measured at the baseline visit, when possible. Information related to HIV care, including nadir CD4 cell counts, and current and past ART history, especially cumulative exposure to first‐generation nucleoside‐reverse‐transcriptase‐inhibitors (NRTIs) (stavudine, zidovudine, didanosine) and non‐nucleoside reverse transcriptase inhibitors (NNRTI) (efavirenz, nevirapine) and protease inhibitors (lopinavir/ritonavir, atazanavir), were extracted from participant medical records.

VCTE was performed using FibroScan^®^ (EchoSens, Paris, France), either M or XL probe where appropriate, by experienced operators in each site following a validated procedure. The final results were expressed as a median of 10 valid measurements. Liver stiffness measurement (LSM) and controlled attenuation parameter (CAP) were used to determine liver fibrosis and steatosis, respectively. VTCE measures were considered reliable when all the following criteria were fulfilled: (i) ≥ 10 successful measurements; (ii) an interquartile range (IQR) lower than 30% of the median value of LSM for fibrosis and CAP for steatosis; and (iii) a success rate of more than 60% [29]. Participant measurements that did not meet these criteria were excluded from the analysis. Significant liver fibrosis was defined as LSM ≥7.1 kPa (METAVIR F ≥2) [30]. The presence of liver steatosis was identified by a CAP ≥248 dB/m which corresponds to steatosis involving at least 5% of hepatocytes [16].

Participants were excluded from this analysis if missing data on any of the following variables: BMI, T2DM, alcohol consumption, HBs antigen, anti‐HCV antibodies, HIV viral load, CAP median and LSM median.

### 2.3 Statistical analysis

Categorical variables were reported as absolute number (*n*) and frequency (%), and continuous variables as median and IQR, overall and stratified by IeDEA region. Univariable logistic regression models were used to determine factors associated with significant liver fibrosis and liver steatosis. Factors with a *p*‐value <0.20 were considered significant and used to build the multivariable models. Risk factors found to be insignificant in the univariable analysis and known in the literature to be associated with liver fibrosis were retained in the multivariable models. A backward stepwise procedure was performed to obtain the multivariable final models. Adjusted odds ratio (OR) estimates were reported with their 95% confidence intervals (95% CI). A *p*‐value of ≤ 0.05 was considered statistically significant. The population attributable fraction (PAF) of significant liver fibrosis for a given etiologic condition was calculated using the following Levin's formula [31]: PAF = p × (RR−1) / p × (RR−1) + 1. In this formula, “p” refers to the prevalence of the etiologic factor in our study population not affected by liver fibrosis. The RR corresponds to relative risk of liver fibrosis associated with a given etiologic factor. The RR has been approximated through the computed OR obtained from the multivariable regression models. All statistical analyses were performed using R software Version 4.2.0.

### 2.4 Ethical considerations

All participants were informed about potential benefits and harms related to their study participation and provided written informed consent prior to being included. The Institutional review boards at each site approved the study protocol (INI‐Fiocruz, Brazil: 28609820.9.0000.5262; CMSDS, Côte d'Ivoire :195‐21; BJ Medical College, India: 00241024; AMPATH Eldoret, Kenya: 0003638; INCMNSZ, Mexico: 3708; Kicukiro Health Center, Rwanda: 885/RNEC/2022; EVT Clinic, Togo: 01/2022/CBRS; CIDRZ, Zambia; 00001131 of IORG0000774.

## 3 RESULTS

**Figure 1:**
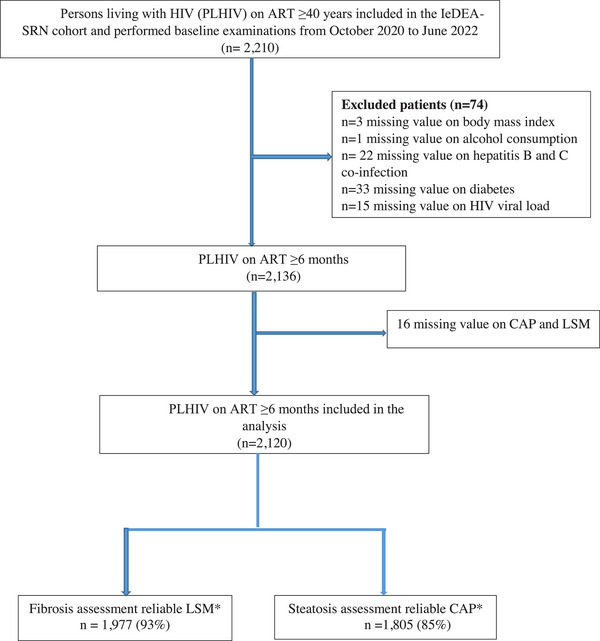
Study flowchart of PLHIV enrolled in the SRN cohort assessed for liver fibrosis and steatosis at baseline visit.

A total of 2210 PLHIV were enrolled in the SRN cohort and had a baseline visit from October 2020 to July 2022. Participants were excluded from this analysis due to missing data on BMI (*n* = 3), alcohol consumption (*n* = 1), T2DM (*n* = 33), hepatitis B and C co‐infection (*n* = 22), HIV viral load (*n* = 15), or missing value on CAP median or LSM median (*n* = 16) (Figure 1). Overall, 2120 PLHIV with a median age of 50 (IQR 45−56) years were initially considered for the analyses. The majority (56%) of participants were females, with significant differences between participating countries (*p*<0.001). HIV sites from sub‐Saharan African countries had higher proportions of female participants compared to sites in India, Brazil and Mexico (Table 1). Over half of the participants were overweight or obese, 32% and 19%, respectively. Approximately half of our participants (53%) had dyslipidaemia, whereas 12% had T2DM and 29% had hypertension, with marked differences according to contributing sites (*p*<0.001) (Table 1). Ninety‐six (4.5%, 95 CI 3.6−5.4) participants were HBsAg positive, ranging from 9.0% in Côte d'Ivoire and Togo to 1.0% in India. Among the 96 participants with positive HBsAg, 82 (85%) were on tenofovir‐based ART, 64% had been previously screened for HBsAg and 43% had HBV infection recorded in their medical record prior to the study visit. Thirty‐seven participants were anti‐HCV positive, with a prevalence of 1.7% (95 CI 1.1−2.3), and ranging from 7.3% in Brazil and Mexico to 0.5% in India. Of the 37 anti‐HCV antibody‐positive participants, 46% had been previously treated for hepatitis C. Of the 29 with HCV RNA testing available, 10 had a detectable HCV RNA with a median value of 6.4 (IQR 5.6–6.7) log UI/ml. Hazardous alcohol use was found in 255 (12%) participants, with important regional differences (*p*<0.001); 26% in Zambia, 16% in Brazil and Mexico, 12% in Côte d'Ivoire and Togo, and 2.5% in India (Table 1).

**Table 1:** Characteristics of PLHIV on ART according to contributing regions (*n* = 2120)

	Asia Pacific (*N* = 197)	Central Africa (*N* = 573)	CCASAnet (*N* = 395)	East Africa (*N* = 190)	Southern Africa (*N* = 184)	West Africa (*N* = 581)	Total (*N* = 2120)
***Age (IQR) (years)***	48 (45, 55)	51 (46, 56)	51 (45, 59)	47 (43, 52)	50 (44, 54)	50 (45, 56)	50 (45, 56)
***Sex at birth***							
Male	104 (52.8)	248 (43.3)	284 (71.9)	55 (28.9)	66 (35.9)	182 (31.3)	939 (44.3)
Female	93 (47.2)	325 (56.7)	111 (28.1)	135 (71.1)	118 (64.1)	399 (68.7)	1181 (55.7)
***Body mass index (kg/m^2^)***							
<25	138 (70.1)	341 (59.5)	145 (36.7)	91 (47.9)	93 (50.5)	231 (39.7)	1039 (49.0)
25.0−29.9	48 (24.4)	152 (26.5)	151 (38.2)	67 (35.3)	59 (32.1)	205 (35.3)	682 (32.2)
≥30	11 (5.5)	80 (14.0)	99 (25.1)	32 (16.8)	32 (17.4)	145 (25.0)	399 (18.8)
***Central obesity*** ^a^							
No	67 (34.0)	298 (52.0)	135 (34.2)	61 (32.1)	78 (42.4)	201 (34.6)	840 (39.6)
Yes	130 (66.0)	275 (48.0)	260 (65.8)	129 (67.9)	106 (57.6)	380 (65.4)	1280 (60.4)
***Hypertension***							
No	150 (87.8)	460 (80.3)	253 (64.1)	138 (72.6)	129 (70.1)	380 (65.4)	1510 (71.2)
Yes^b^	47 (23.9)	113 (19.7)	142 (35.9)	52 (27.4)	55 (29.9)	201 (34.6)	610 (28.8)
***Type 2 diabetes mellitus*** ^c^							
No	154 (78.2)	531 (92.7)	316 (80.0)	179 (94.2)	166 (90.2)	523 (90.0)	1869 (88.2)
Yes	43 (21.8)	42 (7.3)	79 (20.0)	11 (5.8)	18 (9.8)	58 (10.0)	251 (11.8)
***Dyslipidaemia***							
No	101 (51.3)	264 (46.1)	150 (38.0)	19 (10.0)	38 (20.7)	263 (45.3)	835 (39.4)
Yes	89 (45.2)	302 (52.7)	242 (61.3)	82 (43.2)	92 (50.0)	317 (54.6)	1124 (53.0)
Missing	7 (3.6)	7 (1.2)	3 (0.8)	89 (46.8)	54 (29.3)	1 (0.2)	161 (7.6)
***ALT (IQR)***	21 (15.5, 29)	21 (16.0, 28)	28 (21.0, 40)	20 (15.6, 25)	23 (17.0, 30)	20 (16.0, 27)	22 (16.8, 30)
***AST (IQR)***	22 (19.4, 29)	28 (23.0, 34)	23 (19.3, 29)	25 (21.4, 29)	29 (23.0, 36)	28 (23.0, 34)	26 (21.0, 33)
***Positive HBsAg***							
No	195 (99.0)	549 (95.8)	386 (97.7)	188 (98.9)	177 (96.2)	529 (91.0)	2024 (95.5)
Yes	2 (1.0)	24 (4.2)	9 (2.3)	2 (1.1)	7 (3.8)	52 (9.0)	96^d^ (4.5)
***HCV infection***							
Anti‐HCV antibodies−	197 (100.0)	570 (99.5)	366 (92.7)	190 (100.0)	184 (100.0)	576 (99.1)	2083 (98.3)
Anti‐HCV antibodies+/HCV RNA−	0 (0.0)	2 (0.3)	15 (3.8)	0 (0.0)	0 (0.0)	2 (0.3)	19 (0.9)
Anti‐HCV antibodies+/HCV RNA+^e^	0 (0.0)	1 (0.2)	6 (1.5)	0 (0.0)	0 (0.0)	3 (0.5)	10 (0.5)
***Nadir CD4^+^ T‐lymphocyte count***	185 (101.0, 277)	182 (94.2, 284)	171 (61.0, 292)	174 (108.0, 284)	228 (126.0, 366)	190 (96.0, 316)	185 (95.0, 300)
Missing	3	3	0	7	8	90	111
***CD4^+^ T lymphocyte count*** ^f^	516 (339, 692)	534 (389, 691)	651 (442, 858)	409 (273, 600)	516 (377, 658)	546 (378, 739)	540 (374, 729)
***HIV RNA viral load***							
Suppressed	189 (95.9)	517 (90.2)	385 (97.5)	148 (77.9)	183 (99.5)	552 (95.0)	1974 (93.1)
Unsuppressed^g^	8 (4.1)	56 (9.8)	10 (2.5)	42 (22.1)	1 (0.5)	29 (5.0)	146 (6.9)
***AUDIT score***							
<8^h^	192 (97.5)	521 (90.9)	329 (83.3)	179 (94.2)	136 (73.9)	508 (87.4)	1865 (88.0)
≥8	5 (2.5)	52 (9.1)	66 (16.7)	11 (5.8)	48 (26.1)	73 (12.6)	255 (12.0)
***ART exposure at enrolment (IQR)*** ^i^							
Stavudine exposure, months	47 (30, 72)	46 (26, 71)	43 (16, 65)	46 (24, 77)	41 (3, 52)	13 (9.0, 27)	40 (14, 63)
Didanosine exposure, months	0 (0, 0)	62 (62, 62)	25 (13, 49)	24 (18, 32)	45 (45, 45)	23 (16, 24)	25 (13, 49)
Zidovudine exposure, months	89 (42, 126)	145 (78, 175)	77 (29, 125)	66 (16, 104)	77 (47, 97)	91 (34, 107)	93 (38, 125)
Efavirenz exposure, months	50 (19, 71)	70 (28, 126)	90 (37, 140)	58 (22, 88)	68 (46, 82)	30 (13, 67)	57 (21, 97)
Nevirapine exposure, months	96 (54, 147)	163 (119, 190)	18 (9, 42)	97 (28, 141)	73 (40, 97)	90 (36, 118)	114 (49, 158)
Lopinavir/ritonavir exposure, months	0 (0, 0)	52 (41, 64)	51 (28, 110)	66 (15, 94)	61 (25, 91)	51 (11, 98)	53 (23, 94)
Atazanavir exposure, months	54 (38, 74)	42 (24, 75)	60 (21, 108)	50 (29, 55)	0 (0, 0)	9 (5, 13)	33 (11, 74)

^a^ Assessed with waist circumference (WC): sub‐Saharan African: males—WC ≥94 cm is high WC/females—WC ≥80 cm is high WC; South East Asia and Southern America: males—WC ≥90 cm is high WC/females—WC ≥80 cm is high WC.

^b^ Systolic BP ≥140 mmHg and/or diastolic BP ≥ 90 mmHg or being prescribed anti‐hypertension medication.

^c^ Assessed with plasma glucose and HbA1c or history of diabetes treatment.

^d^ 85.4% were on TDF, 83.3% were on lamivudine, 64.6% were screened for HBsAg, 42.7% had an HBV infection history and 12.6% have been vaccinated for HBV infection.

^e^ Eight missing data.

^f^ Ten missing data.

^g^ >1000 copies/ml.

^h^ <7 for females.

^i^ Cumulative exposure to the listed ART regimens at SRN enrolment visit.

The median IQR CD4 cell count was 540 [IQR 374−729] cells/mm^3^ and 146 (6.9%) participants had an unsuppressed HIV viral load (>1000 copies/mm^3^) at the study visit. Based on ART regimen records, 1063 (50%) of participants were previously exposed to zidovudine for a median duration of 93 months [IQR 38−125], 545 (26%) participants were treated with a stavudine‐containing regimen for a median duration of 40 months [IQR 14−63]. Exposure to didanosine‐containing ART was observed in 81 (3.8%) participants with a median duration of 25 months [IQR 13−49]. A total of 1426 (67%) participants were exposed to efavirenz drugs with a median duration of 57 months [IQR 21−97]. Exposure to atazanavir‐containing ART was found on 385 (18%) participants, with a median duration of 93 months [IQR 38−125] (Table 1). For participants treated with didanosine and stavudine, the median time since discontinuation was 20 years [IQR 13−24] and 12 years [IQR 11−13], respectively.

**Figure 2:**
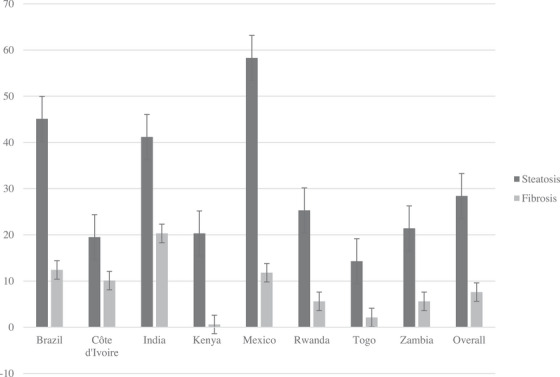
Prevalence of liver steatosis and fibrosis assessed by transient elastography according to participating sites.

Measurements of LSM and CAP for liver fibrosis and steatosis assessments were considered reliable for 93% (*n* = 1977) and 85% (*n* = 1805) of the participants, respectively. Median LSM and CAP were 4.7 [IQR 3.9−5.6] kPa and 220 [IQR 191−253] dB/m, respectively. Significant liver fibrosis was present in 151 (7.6% [95% CI, 6.4−8.8]) participants, and varied significantly in prevalence between sites (*p*<0.001). Compared to sites in sub‐Saharan Africa, higher prevalence of liver fibrosis was found in India (20%; 95% CI 14−26), Brazil (12%; 95% CI 7.9−17) and Mexico (12%; 95% CI 6.6−17). A total of 512 (28% [95% CI, 26−31]) participants had liver steatosis, with higher prevalence in India (41%; 95% CI 33−49); Brazil (45%, CI 38−52); and Mexico (58%, 95% CI 50−66) (Figure 2).

### 3.1 Factors associated with significant liver fibrosis

**Figure 3:**
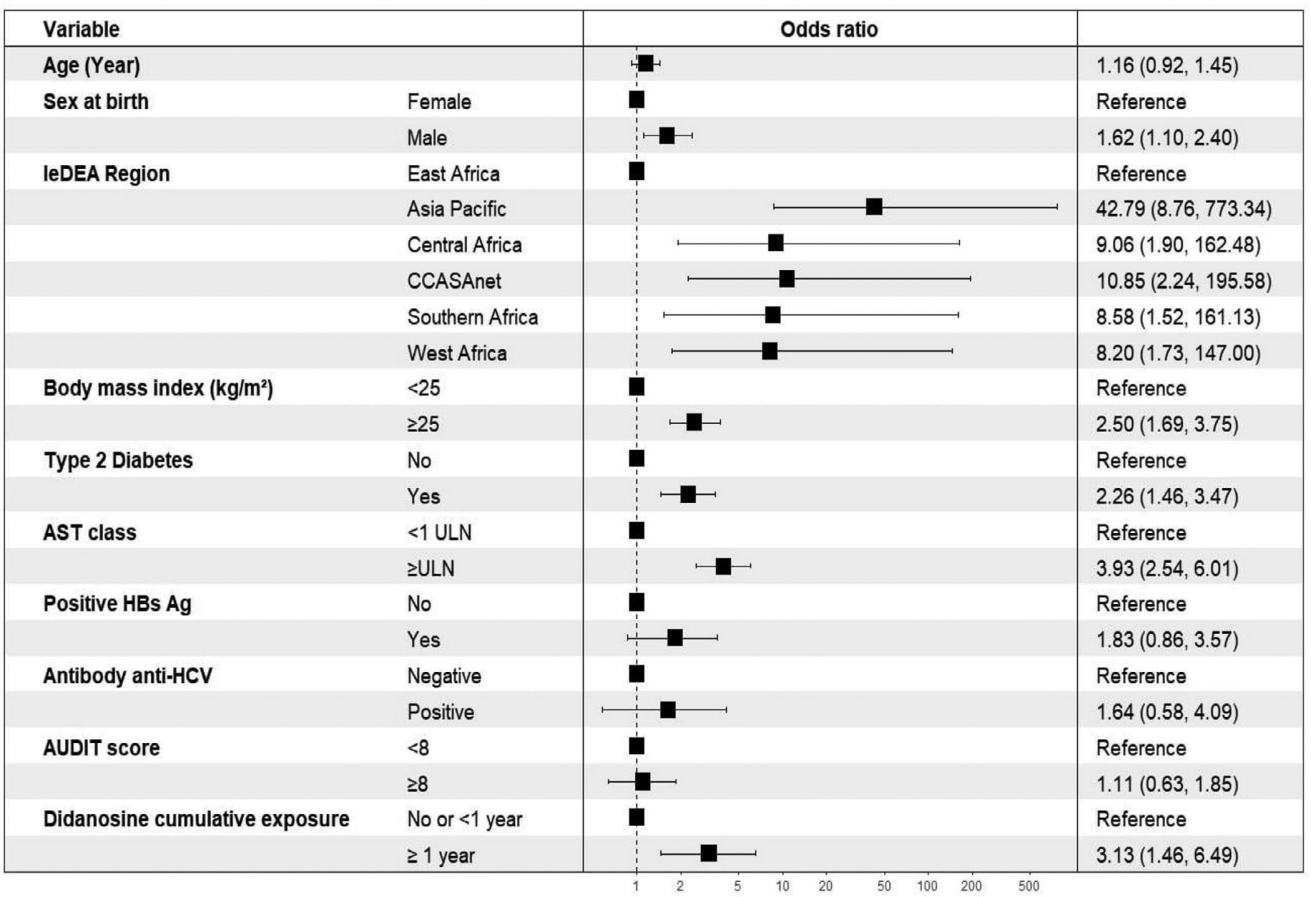
Factors associated with significant liver fibrosis in adults living with HIV on ART.

In multivariable analysis, the following factors were independently associated with significant liver fibrosis: male sex at birth (OR 1.62, 95% CI 1.10−2.40), overweight/obesity (BMI ≥25 vs. BMI <25 kg/m^2^) (OR 2.50, 95% CI 1.69−3.75) and T2DM (OR 2.26, 95% CI 1.46−3.47). Elevated AST measure (≥ 1 × ULN vs. <1 × ULN) (OR 3.93, 95% CI 2.54−6.01) and a past exposure to didanosine‐containing ART for ≥ 1 year (OR 3.13, 95% CI 1.46−6.49) (ref: No exposure or exposure <1 year) were also associated with significant liver fibrosis. Hazardous alcohol use (OR 1.11, 95% CI 0.63−1.85) as well as positive HBsAg (OR 1.83, 95% CI 0.86−3.57) or positive anti‐HCV antibodies (OR 1.64, 95% CI 0.58−4.09) were not associated with significant liver fibrosis in this study (Figure 3). The PAF of liver fibrosis for overweight/obesity and T2DM were 42% and 11%, respectively. The PAF of liver fibrosis was only 3% for positive HBsAg, 1% for both positive anti‐HCV antibodies and hazardous alcohol use (Table 2).

**Table 2:** Levin's PAF of liver fibrosis attributed by overweight/obesity, diabetes mellitus, hazardous alcohol use, positive HBsAg and positive anti‐HCV antibody

	Prevalence^a^, %	OR (95% CI)	PAF, %
Overweight/obesity (BMI ≥25 kg/m^2^)	49%	2.50 (1.69, 3.75)	42%
Diabetes mellitus	10%	2.26 (1.46, 3.47)	11%
Hazardous alcohol use (AUDIT score ≥8^b^)	12.%	1.11 (0.63, 1.85)	1.0%
Positive HBsAg	4.5%	1.83 (0.86, 3.57)	3.0%
Positive antibody anti‐HCV	1.6%	1.64 (0.58, 4.09)	1.0%

Abbreviations: CI, confidence interval; OR, odds ratio; PAF, population attributable fraction.

^a^ Prevalence of the etiologic factor in our study population not affected by liver fibrosis.

^b^ <7 for females.

### 3.2 Factors associated with liver steatosis

**Figure 4:**
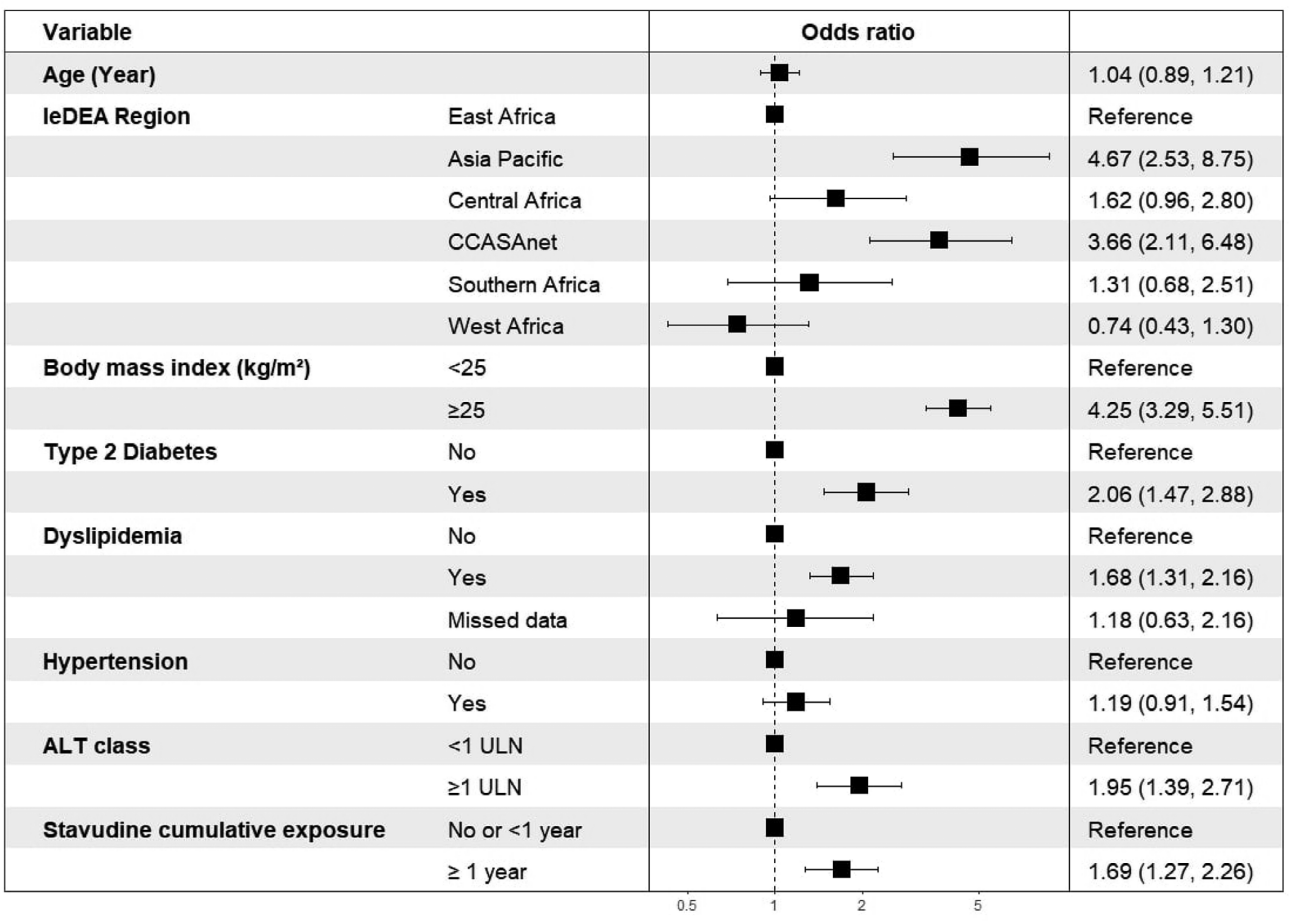
Factors associated with significant liver steatosis in adults living with HIV on ART.

In multivariable analysis; BMI ≥25 kg/m^2^ (OR 4.25, 95% CI 3.29−5.51) (ref: BMI<25 kg/m^2^), T2DM (OR 2.06, 95% CI 1.47, 2.88), dyslipidaemia (OR 1.68, 95% CI 1.31, 2.16) as well as elevated ALT (≥ 1 × ULN) (OR 1.95, 95% CI 1.39−2.71) were independently associated with the presence of liver steatosis. Participants who were exposed to a stavudine‐containing regimen for ≥1 year were more likely to have liver steatosis than those not exposed to stavudine or treated for <1 year (OR 1.69, 95% CI 1.27, 2.26). In addition, those with hypertension had a trend towards liver steatosis (OR 1.19, 95% CI 0.91, 1.54) (Figure 4).

## 4 DISCUSSION

We found a relatively high prevalence of significant liver fibrosis and steatosis in this population of adults living with HIV on ART from eight LMICs in six global regions, with significant variations between sites. Despite variation in the prevalence of metabolic and infectious risk factors observed across settings, obesity and T2DM, accounted for the majority burden of liver fibrosis, with HBV and HCV contributing marginally.

The substantial variability in the prevalence of liver fibrosis and steatosis among PLHIV across countries and regions is consistent with previous studies in similar settings. In a study performed in Brazil, Perazzo et al. reported prevalence of 9% and 35% for fibrosis (LSM ≥ 8.0 kPa) and steatosis (CAP ≥248 dB/m) in people with HIV and no other concomitant infections, respectively [32]. Results of a study performed in three countries in West Africa (Togo, Côte d'Ivoire and Senegal) showed a prevalence of 5.3% for liver fibrosis using LSM ≥7.1 kPa [18]. Findings of a study in China showed a prevalence of 14% for liver fibrosis using magnetic resonance spectroscopy and LSM ≥7.0 kPa and a prevalence of 29% for liver steatosis [33]. A previous systematic review and meta‐analysis based mostly on studies from high‐income countries reported a prevalence of liver steatosis (based on imaging) and liver fibrosis (≥F2 by liver biopsy) of 35% and 22%, respectively, in people with HIV and no other concomitant infections [34]. Although liver fibrosis and steatosis were important in all sites, the highest prevalence was reported in India, Brazil and Mexico. Our findings are corroborated by findings of a more recent meta‐analysis reporting a higher prevalence of liver fibrosis and steatosis in PLHIV from Asian and South American countries compared to sub‐Saharan African countries [9, 13]. These differences could be partly related to the higher prevalence of metabolic disorders observed in India, Brazil and Mexico, identified as important risk factors for liver steatosis and its progression [13].

While the reported prevalence of liver fibrosis and steatosis in our population may reflect the burden of metabolic disorders in the general population of these LMICs, additional HIV‐related factors should be considered [35]. Indeed, HIV itself and exposure to ART have been linked with metabolic disorders, including T2DM and hyperlipidaemia, as well as a higher risk of liver fibrosis through drug‐induced liver toxicity; all together supporting a higher risk of liver disease in PLHIV [36, 37].

Our study revealed a significant association between male sex at birth and liver fibrosis. Similar results were previously reported in studies from Zambia and Ghana [12, 38]. This association could be explained by the oestrogen release in pre‐menopausal women, which reduces the activation and proliferation of hepatic stellate cells, involved in the liver fibrogenesis process [39, 40]. A protective effect of oestrogen against metabolic conditions in pre‐menopausal women has been also reported in the literature [41]. Other factors such as metabolic disorders have been repeatedly associated with liver disease [34]. Similar to our results, Mohr et al. report a strong association between metabolic disorders, including T2DM and obesity, and significant liver fibrosis (OR = 4.65, 95% CI 1.42–15.19 and OR = 5.47, 95% CI 1.81–16.51, respectively) among PLHIV [42]. A recent study performed by Michel et al. also identified T2DM as an independent predictor of liver fibrosis [43]. Considering these results, PLHIV with T2DM or obesity should be monitored for liver disease, given the rapid progression of fibrosis in diabetic and obese patients with liver steatosis [13, 44].

In addition to metabolic disorders, we observed a significant association of prolonged exposure to didanosine‐containing regimens, with significant fibrosis and prolonged exposure to stavudine and liver steatosis. These associations have been previously reported in high‐income settings [11, 45, 46]. While these NRTIs, known to induce hepatic mitochondrial toxicity and metabolic abnormalities, were replaced by newer NRTIs years ago, previous studies have highlighted the persistence of liver damage years after their withdrawal [47, 48]. Indeed, over three‐quarters of our participants who received didanosine or stavudine had discontinued them over a decade ago. This potential cumulative hepatotoxicity supports the need for a liver monitoring strategy in participants with prolonged exposure to these former hepatotoxic first generation of ART drugs. Modern ART has been recommended as first‐line treatment over the past decade to counterbalance the hepatotoxic effect of these drugs. However, some of those drugs, especially integrase strand transfer inhibitor and tenofovir alafenamide, have been reported to be associated with metabolic disorders, including weight gain, which could favour the development and progression of liver steatosis, and fibrosis [49, 50, 51]. This complex interplay between ART exposure and liver steatosis deserves further attention through a longitudinal approach. The follow‐up of the SRN cohort will allow us to measure the impact of these new ART regimens on liver disease development and its progression over time.

In our study, HBsAg and anti‐HCV were not significantly associated with liver fibrosis, in contrast to previous studies [18, 19]. This could be related to the use of tenofovir disoproxyl fumarate and lamivudine, two molecules active against HBV, in most of our sites. Moreover, anti‐HCV therapy is currently available across several of the countries participating in our project. As active treatments against these infectious conditions are now increasingly scaled‐up in PLHIV, it is crucial to focus on metabolic causes of liver disease. Indeed, by using the PAF, we found that 53% of liver fibrosis cases could potentially be prevented by controlling overweight, obesity and diabetes in the population of PLHIV. In contrast, by tackling HBV, HCV and hazardous alcohol use, the proportion of liver fibrosis development would decrease by only 5% in this population. These results suggest that liver disease‐related morbidity could be significantly reduced by integrating the management of these preventable metabolic risk factors within routine HIV care.

Strengths of this study include using data from a large multicentre cohort study across six global regions and eight LMICs, with a standardized data collection protocol, VCTE examinations performed by experienced operators, and cutoffs to define liver fibrosis and steatosis relied on cutoffs used in previous studies [18, 19, 38]. The current study has some limitations to consider. We were not able to assess several factors potentially leading to liver fibrosis or steatosis, including exposure to aflatoxins, iron overload, exposure to other hepatotoxic drugs, such as anti‐tuberculosis drugs, or the presence of other infectious conditions that could lead to liver fibrosis such as schistosomiasis. PAF estimates assume a causal relationship between each associated factor and liver fibrosis, and that the RR is a good approximation of the true causal effect. Based on this assumption, our PAF estimates may be higher than the true value, as we used OR estimates instead of RR and, considering that OR generally overestimates RR. Further, longitudinal studies are needed to test whether intervention on these exposures reduces the risk of liver fibrosis. Given the cross‐sectional nature of this study, we could not draw formal inferences between liver fibrosis and steatosis, and associated factors, although most reported associations relied on previously documented etiologic pathways.

## 5 CONCLUSIONS

Liver fibrosis and steatosis were common among adults living with HIV on ART in LMICs, representing an important health concern among this population. Metabolic disorders, including overweight/obesity and T2DM, are associated with increased risk of significant liver steatosis and fibrosis, and could be initial targets for liver disease prevention strategies. Furthermore, integrating prevention and care of metabolic risk factors, and dedicated monitoring of liver disease in PLHIV with metabolic diseases should be advocated in resource‐limited settings. However, further prospective studies are needed to confirm the causative nature of our study findings.

## COMPETING INTERESTS

The authors have no competing interests to disclose.

## AUTHORS’ CONTRIBUTIONS

MKP conceptualized and designed the study, statistically analysed and interpreted the data, drafted and critically reviewed the manuscript. AKM, GW, GM, NS, JR, MHK, FS, RW and FM critically reviewed the study design and the manuscript. AL, EM, LD, SS, RM, AA, AM, SG, DR and PA critically reviewed the manuscript. HP and AJ conceptualized and designed the study, supervised the study, interpreted the data and critically reviewed the manuscript. All authors critically reviewed the manuscript and agreed on its final version.

## FUNDING

The International epidemiology Databases to Evaluate AIDS (IeDEA) is supported by the U.S. National Institutes of Health's National Institute of Allergy and Infectious Diseases (Grant numbers: **Asia‐Pacific**, U01AI069907; **CCASAnet**, U01AI069923; **Central Africa**, U01AI096299; **East Africa**, U01AI069911; **Southern Africa**, U01AI069924; **West Africa**, U01AI069919. Informatics resources are supported by the Harmonist project, R24AI24872).

## DISCLAIMER

This work is solely the responsibility of the authors and does not necessarily represent the official views of the institution mentioned above.

## Data Availability

Data are available on reasonable request.
